# Fecal microRNAs, Fecal microRNA Panels, or Combinations of Fecal microRNAs with Fecal Hemoglobin for Early Detection of Colorectal Cancer and Its Precursors: A Systematic Review

**DOI:** 10.3390/cancers14010065

**Published:** 2021-12-23

**Authors:** Zitong Zhao, Anna Zhu, Megha Bhardwaj, Petra Schrotz-King, Hermann Brenner

**Affiliations:** 1 Division of Clinical Epidemiology and Aging Research, German Cancer Research Center (DKFZ), 69120 Heidelberg, Germany; zitong.zhao@dkfz-heidelberg.de (Z.Z.); anna.zhu@dkfz-heidelberg.de (A.Z.); 2 Medical Faculty Heidelberg, University of Heidelberg, 69120 Heidelberg, Germany; 3 Division of Preventive Oncology, German Cancer Research Center (DKFZ) and National Center for Tumor Diseases (NCT), 69120 Heidelberg, Germany; megha.bhardwaj@nct-heidelberg.de (M.B.); petra.schrotz-king@nct-heidelberg.de (P.S.-K.); 4 German Cancer Consortium (DKTK), German Cancer Research Center (DKFZ), 69120 Heidelberg, Germany

**Keywords:** colorectal cancer, miRNA, stool, early detection

## Abstract

**Simple Summary:**

Screening for colorectal cancer is effective for the reduction of both CRC incidence and mortality in the population at average risk. The use of innovative and robust biomarkers to enhance the potential of noninvasive CRC screening remains desirable. We aimed to conduct a systematic literature review on the diagnostic performance of fecal miRNA markers for CRC and its precursors. Several studies have reported quite promising results, in particular by combining fecal miRNA measurements with fecal hemoglobin. However, current evidence is limited by substantial heterogeneity in the methodology from study design to biosample analysis. Our review is intended to provide a valuable reference for future biomarker studies in early colorectal cancer detection. Looking at fecal miRNAs, we draw attention to the various biases to be avoided or at least minimized, by applying a harmonized methodology including true screening settings and comparable sample pre-analytics, as well as the validation of biomarkers.

**Abstract:**

Colorectal cancer (CRC) is the third most common cancer and the second leading cause of cancer mortality globally. Fecal miRNAs have been suggested to be promising biomarkers for CRC early detection. We aimed to conduct a systematic literature review on the diagnostic performance of fecal miRNA markers for CRC and its precursors. PubMed and Web of Science were searched to retrieve relevant articles published up to 7 December 2021. Information on study design, characteristics of study population, pre-analytics (sample collection, processing, and storage), fecal miRNA extraction and quantification technologies, and diagnostic performance (including sensitivity, specificity, and area under the curve (AUC)) were summarized. Twenty studies reporting on 31 individual miRNAs and 16 miRNA panels (with 2–9 markers) for CRC diagnosis were identified. Substantial heterogeneity existed regarding stool sample collection, processing, storage, and miRNA extraction and normalization. For two individual miRNAs and one miRNA panel, values ≥ 80% were reported for both sensitivity and specificity; however, none of these results were either internally or externally validated. In a study among fecal immunochemical test-positive cases recruited from a true screening setting, better diagnostic performance was identified and internally validated for a combination panel including two miRNAs, fecal hemoglobin level, and patient age and sex, compared with fecal hemoglobin concentration alone. Fecal miRNAs or miRNA panels, possibly in combination with fecal hemoglobin test, may be promising candidates for noninvasive CRC early detection. However, large prospective and well-designed studies in CRC screening cohorts are required to validate promising miRNAs or miRNA panels.

## 1. Introduction

Colorectal cancer (CRC) is the third most common cancer and the second leading cause of cancer mortality globally, with 1.9 million incident cases and 935,000 deaths estimated in 2020 [1]. As most CRCs progress slowly from precancerous lesions to malignant tumor over many years, chances of screening and early detection are substantially higher than for most other cancer types. It has been shown that the disease burden can be effectively reduced with population-based screening [2,3]. Currently established CRC screening strategies fall into two categories: stool-based tests (high-sensitivity guaiac fecal occult blood test (gFOBT), fecal immunochemical tests (FIT), stool DNA-FIT test, etc.), and direct visualization tests (flexible sigmoidoscopy, colonoscopy, etc.) [4]. Colonoscopy is the gold-standard for reliable early detection of CRC and its precursors, but its use as primary screening examination in population-based screening is hampered by its invasive nature, limited capacities, low compliance, operator dependence, and high cost [5,6,7,8]. Compared to colonoscopy, flexible sigmoidoscopy is less invasive and costly, and there is no need for complete bowel cleansing and sedation, but it does not visualize neoplasms in the proximal colon [3,9]. While FIT is widely used as an effective noninvasive method for early detection of CRC in a gradually increasing number of countries [10], it has substantially lower sensitivity for detecting advanced adenomas (AA) and stage-I CRC [11,12]. Thus, the use of innovative biomarkers to enhance the potential of noninvasive CRC screening remains desirable.

MicroRNAs (miRNAs) are a class of noncoding single stranded RNAs composed of 18–22 nucleotides, with regulatory and catalytic functions [13]. Aberrant miRNAs are associated with the development of various cancer types [14,15], including CRC [16,17,18]. These alterations in tumors are also mirrored in biofluids, and have been detected in blood, urine, and stool [19,20,21]. Importantly, it was demonstrated that extracellular miRNAs are highly stable, resisting ribonuclease degradation at room temperature up to 24 h in plasma [22] and to 72 h in stool [23], providing the rationale for using miRNAs as noninvasive and robust clinical biomarkers for cancer early detection. Recently, the possibility of using miRNAs in stool as a non-invasive detection method for CRC has received increasing attention. In this systematic review, we aim to provide a comprehensive overview of studies that assessed the diagnostic value of fecal miRNAs for CRC and its precursors.

## 2. Materials and Methods

This systematic review was conducted in accordance with Preferred Reporting Items for Systematic Reviews and Meta-Analyses protocols (PRISMA-P) [24]. This study was registered in the Research Registry (London, UK) (reviewregistry1259).

### 2.1. Data Sources and Search Strategy

Scientific citation databases of PubMed and Web of Science (WOS) were searched for relevant studies from the inception to 7 December 2021. The search items included: (colorectal OR colon OR colonic OR rectal OR rectum) AND (carcinoma OR neoplasm OR adenocarcinoma OR cancer OR tumor OR tumour * OR malignant * OR adenoma *) AND (Stool OR fecal OR faecal OR feces OR faeces) AND (“micro RNA*” OR microRNA * OR miRNA * OR miR *). The asterisk (*) here represents any group of characters, including no character. Reference sections of identified publications were also checked to find additional relevant studies.

### 2.2. Eligibility Criteria

Studies that reported measures of diagnostic value of fecal microRNAs for early detection of CRC or colorectal adenoma (CRA) were included. Firstly, the articles were pre-selected by reviewing the title and abstract. Articles were excluded if they were: (1) duplicates, (2) non-English studies, (3) not original studies, (4) not full papers, (5) not human studies, (5) not related to the topic. Then, the full texts of remaining articles were reviewed, and the studies that did not report key study characteristics and diagnostic performance indicators of microRNA markers (such as sample size, sensitivity, specificity, or area under the curve (AUC)) were excluded.

### 2.3. Data Extraction and Quality Assessment of Each Study

Two authors (Z.Z. and A.Z.) independently read and extracted data from included studies. The following variables were extracted from each study: first author, year of publication, study population (country, numbers of cases and controls, age, sex distribution, and tumor stage and location for cases), study design, sample collection, processing, and storage, microRNA measurement method, identified microRNAs, and indicators of diagnostic performance (including sensitivity, specificity, AUC, and *p*-value). Information on fecal hemoglobin was also extracted if provided. Results for individual miRNAs with *p*-value > 0.05 are not shown.

The Quality Assessment of Diagnostic Accuracy Studies 2 (QUADAS-2) [25] instrument was applied to assess the quality of each included study with respect to patient selection, index test, reference standard, and flow and timing. In QUADAS-2, each domain is evaluated for risk of bias, and the first three domains are evaluated for applicability. The risk of bias and concerns regarding applicability for each study were rated as “High”, “Low”, or “Unclear”. The above-mentioned two authors (Z.Z., A.Z.) performed quality assessment independently utilizing the software Review Manager 5.4.1 (The Cochrane Collaboration, London, UK, 2020).

## 3. Results

### 3.1. Literature Search Result

**Figure 1:**
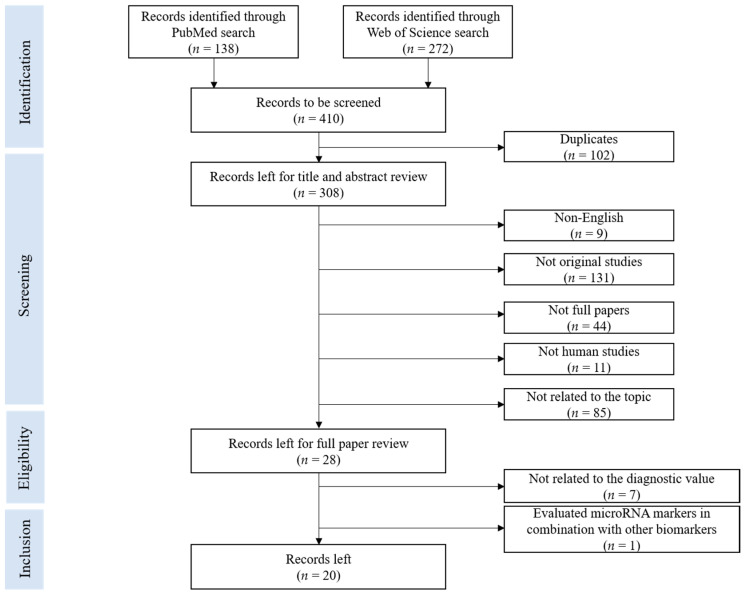
PRISMA flow diagram for literature search process for records identified via PubMed and Web of Science database.

The literature search in the above-mentioned databases using the aforementioned search terms yielded 410 records. Details of the selection process are presented in the PRISMA flow diagram (Figure 1). Upon application of eligibility criteria, 28 articles were selected for full review. Eight articles were further excluded, because they did not provide relevant data of diagnostic performance or because they looked exclusively at the combination of miRNAs with other types of biomarkers. No additional studies were identified by cross-referencing. Finally, 20 studies on diagnostic performance of fecal microRNA published up to 7 December 2021 were included in this systematic review. Data on diagnostic performance of individual fecal miRNAs and miRNAs panels for CRC and CRA are summarized. Extracted information on key study characteristics, details of miRNA detection techniques, stage-specific results, location-specific results, the PRISMA checklist, and the risk of bias of individual studies are reported in the Supplementary Files (Tables S1–S4, and Figures S1 and S2).

### 3.2. Study Characteristics

Key characteristics of all included studies are shown in Table S1. Sixteen studies were conducted in Asia (16/20), of which nine were from China [23,26,27,28,29,30,31,32,33], three from Iran [34,35,36], two from Japan [37,38], one from Singapore [39], and one from Korea [40]. Only one study was carried out in the US [41], and the remaining studies were conducted in Europe—two from Italy [42,43] and one from Spain [19]. The studies were almost exclusively conducted in clinical settings comparing clinically diagnosed cases with controls. The single study explicitly reporting inclusion of cases selected in a true CRC screening setting recruited only FIT-positive individuals [19]. Healthy controls from a colonoscopy screening program were recruited in two studies [19,23]. Eight studies (8/20) recruited CRAs as an individual case group [19,23,26,27,28,29,32,41], but only six of them reported diagnostic performance indicators specifically for this case group. Numbers of participants ranged from 17 [39] to 198 [27,29] for CRC cases, from 20 [26] to 483 [19] for CRA cases, and from 16 [35] to 247 [30] for controls. Among 20 studies, only 17 performed colonoscopy for all controls, of which 14 (14/17) explicitly defined people with no findings as healthy controls. Male participants were overrepresented in 15 studies, and two studies did not report age and gender distribution of the study participants [26,33]. Nineteen studies (19/20) reported the stage distribution of CRC cases, and two of them included only early-stage cases [34,35].

### 3.3. Fecal miRNA Detection Methods

All twenty studies quantified miRNA levels using reverse transcription polymerase chain reaction (RT-PCR). However, there were great variations in the methodology from miRNA extraction to quantification (Table S1). For miRNA normalization, absolute quantitation was applied in five studies for fecal miRNA [19,23,27,28,29]. Although internal control miRNAs varied in the remaining 15 studies, U6 snRNA was most commonly used (8/15) [26,31,32,33,34,35,37,40]; others included miR-16 [33,36], miR-200b-3p [41], cel-miR-238 [30], miR-16-3p [43], miR-1202 [39], miR-4257 [39] miR-24 [38], and miR-378 [42]. The studies also used different amounts of stool samples (50–500 mg) and kits for miRNA extraction including miRNeasy mini kit (Qiagen) (14/20), mirVanaTM miRNA isolation kit (2/20), TRIzol (2/20), stool RNA extraction kit (Omega) (1/20), and Stool Total RNA purification kit (1/20). Additionally, only three studies used preservation buffers for sample transportation to the laboratory before freezing, including FIT buffer (OC-Sensor, Eiken Chemical, Tokyo, Japan) [19,30] and EDTA buffer [41].

### 3.4. Diagnostic Performance of Fecal miRNA Markers for Detection of Colorectal Neoplasms

Nineteen studies, conducted in clinical settings, included 26 miRNAs and evaluated the diagnostic performance (AUC, sensitivity, specificity) of individual fecal miRNAs on CRC (Table 1). Reported sensitivities and specificities for individual fecal miRNAs ranged from 15 [37] to 97% [33] and 48 [40] to 100% [39], respectively. AUC was reported in 16 studies, and the values ranged from 0.64 [23] to 0.97 [39]. *p*-values for the statistical significance of AUC values were stated in five studies, ranging from 0.017 to <0.0001. 

Eight of the 19 studies reported diagnostic performances of miRNA panels (Table 2). None of these studies performed internal or external validation for correction of overoptimism. Eighteen different miRNAs were evaluated in 15 miRNA panels, and the number of miRNAs in each panel ranged from 2 to 9. The sensitivity and specificity of miRNA panels ranged from 46 [37] to 97% [40] and 38 [40] to 95% [41], respectively. AUCs were reported by five studies, and ranged from 0.75 [27] to 0.89 [41]. Koga et al. [38] investigated the usefulness of fecal miR-106a to detect CRC patients with false-negative FIT results. In this study, the combination of fecal miR-106a with FIT resulted in a better sensitivity (71%) and a similarly stable specificity (96%) than FIT alone (61% sensitivity, 98% specificity).

Diagnostic values for CRA were determined on six individual miRNAs and four miRNA panels in six studies (Table 3 and Table 4), one of which included only AA cases [41]. miR-21 (85% sensitivity, 63% specificity) was reported having high sensitivity for CRA detection [32]. A combination panel (fecal level of miR-421, miR-27a-3p, hemoglobin, and patient age and sex) [19] showed a slightly improved AUC of 0.64 (49% sensitivity, 71% specificity) identifying patients with AA, compared with an AUC of 0.59 (43% sensitivity, 63% specificity) for fecal hemoglobin concentration alone (Table 4). 

Duran-Sanchon et al. [19], the only study recruiting FIT-positive participants from a true CRC screening program, reported the diagnostic value of seven individual miRNAs and one miRNA-based panel for CRA and CRC (Table 4). All AUCs were adjusted for age and sex. This study identified and internally validated (10-fold cross-validation) a better AUC of 0.93 (97% sensitivity, 43% specificity) for a combination panel (miRFec algorithm: fecal level of miR-421, miR-27a-3p, hemoglobin, and patient age and sex) identifying patients with CRC, compared to fecal hemoglobin concentration alone with an AUC of 0.67 (100% sensitivity, 31% specificity). The team further delineated four risk categories for all participants using miRFec algorithm scores [44] and found that the scores were independently associated with the presence of advanced neoplasia (AA and CRC) (*p* < 0.001). Subjects in the highest category (scores > 3.09) were 8-fold more likely to have advanced neoplasia than subjects in the lowest category (scores < 2.14).

Seven studies (7/20) assessed the stage-specific diagnostic values [23,27,28,33,36,37,41] (Table S2). Two studies observed distinguishing ability in differentiating early stage from late stage, for miR-135b-5p (AUC = 0.92, *p* = 0.0022) [33], and miR-21 (AUC = 0.87) [36], with higher miRNA expression levels in advanced stage cases than in early-stage cases. Among seven studies, no significant difference in sensitivity was found in early-stage and late-stage CRC detection. Six studies (6/20) reported a tumor-location-specific diagnostic value [23,27,28,29,37,41] (Table S3), and three of them showed better sensitivities of miRNAs and miRNA panels in distal lesions than in proximal lesions, including miR-92a (*p* = 0.01), Panel L (miR-17-92 cluster, miR-21, miR-135, *p* = 0.0001), Panel M (miR-17-92 cluster, *p* = 0.001), and Panel O (miR-144-5p, miR-451a, *p* = 0.0084) [23,37,41].

Among 31 miRNAs, ten (miR-21, miR-92a, miR-20a, miR-223, miR-144-5p, miR-135b, miR-18a, miR-29a, miR-451, and miR-221) were reported to be significantly associated with CRC in at least two studies (Table 5). Two of these 10 miRNAs (miR-223, and miR-29a) showed contradictory dysregulation direction. All included studies described the direction of dysregulation of miRNAs in stool. MiR-21 was the most frequently reported miRNA in five studies [23,32,36,37,40], mainly in miRNA panels.

### 3.5. Assessment of Risk of Bias across Studies

The results for the quality assessment of all reviewed studies using QUADAS-2 were summarized in Figures S1 and S2. Any initial inconsistencies were resolved by further discussion between the investigators. The greatest potential risk of bias and applicability concerns came from study participant selection, as only one study [19] recruited participants from a true screening program while all other studies recruited patients already diagnosed in clinical settings. For 16 out of 20, the “index test” domain presented unclear risk of bias because it was unclear whether the index test results were gained and interpreted without knowledge of the colonoscopy results. Sixteen of 20 studies presented low risk of bias in “reference standard” and “flow and timing” domains, and further rated of low concern in “reference standard” and “index test” because the implementation and interpretation of “index test” were consistent with the review question.

## 4. Discussion

This review provides an overview of studies reporting on fecal single miRNAs, miRNA panels, or combinations of fecal miRNAs with fecal hemoglobin for the detection of CRC and its precursors. Overall, 20 papers published from 2010 to 2021 and reporting on 31 individual miRNAs and 16 miRNA panels were identified. A broad range of values for diagnostic performance indicators were reported, with AUCs, sensitivities, and specificities ranging from 0.64 to 0.97, 15% to 97%, and 38% to 100%, respectively. Better diagnostic performance was reported for a combination of fecal miRNAs with fecal hemoglobin level compared with fecal hemoglobin or fecal miRNAs alone. However, substantial heterogeneity existed regarding study settings and pre-analytics steps, which require careful consideration in the interpretation of results.

Unlike blood in stool, fecal miRNAs in consistently exfoliating colonocytes are highly reproducible, which appears to lead to similar test results in repeated stool sampling [23]. With relatively high stability and reproducibility in stool [23,46], fecal miRNAs are regarded as promising biomarkers for non-invasive CRC screening. Intestinal epithelial cells are believed to be the main source of fecal miRNAs [47]. However, Phua et al. [39] reported varying degrees of influence of fecal blood on the levels of fecal miR-451, miR-223, and miR-135b. For example, as observed in this systematic review, the level of fecal miR-451 increased significantly in the presence of blood even at low concentrations (0.1 mg Hb/g stool), and miR-451 achieved a high diagnostic performance for CRC with an AUC of 0.97 (88% sensitivity, 100% specificity). Wu et al. [41] used erythrocyte-specific miRNA markers to discriminate between blood and colonocytes, yielding an AUC of 0.89 (66% sensitivity, 95% specificity) for CRC detection. To assess the potential overlap of circulating miRNAs and fecal miRNAs found to be related with CRC, we retrieved a list of circulating miRNAs (*n* = 91) for CRC detection from Raut et al. (2020) [20]. Nineteen miRNAs were reported to be related with CRC in both fecal and serum/plasma samples (Figure S3). These findings may indicate the presence of additional factors, beyond those of colonocytes contributing to the diagnostic performance of fecal miRNAs, pointing to a potential role of blood-borne miRNAs in stool. 

In total, there were five individual miRNAs (miR-29a, miR-21, miR-135b-5p, miR-451, and miR-92a) and four miRNA panels (Panel A, J, N, and P) for which sensitivities for CRC detection above 80% were reported, with corresponding specificities ranging from 52–100% and 33–82%, respectively. Sensitivities and specificities of at least 80% were observed for miR-21 [36], miR-451 [39], and Panel J (miR-21, miR-146a) [32]. However, these impressive results were not validated further and might be partly attributed to overestimation and overoptimism. This underlines the importance of internal and external validation in the assessment of diagnostic biomarkers [48]. In the context of a true screening setting, Duran-Sanchon et al. [19,44] reported a significant improvement using an miRNA-based algorithm (fecal level of miR-421, miR-27a-3p, hemoglobin, and patient age and sex) for CRC detection rather than assaying for fecal hemoglobin concentration alone [19]. Although including only FIT-positive individuals for evaluation, this study enabled assessment of the possibility of using the miRFec algorithm to decrease unnecessary work-up colonoscopies for a subset of FIT-positive participants in a true CRC screening setting. At 50% specificity within the group of FIT positives, 34% of colonoscopies were avoided with 97% sensitivity for CRC and 79% for advanced neoplasia [44]. Previous studies have also reported that many individual miRNAs are not specific to a single disease but usually observed in various pathologies [49]. For example, miR-21, the most frequently reported one in our study, might be a general disease marker, for not only cancer but also non-cancer diseases [50]. In comparison to single miRNAs, complex miRNA panels seem to be more disease-specific [49]. In general, the combination of fecal miRNA panel with fecal hemoglobin concentration might be a promising approach to improve the efficiency and effectiveness of FIT-based screening programs and, consequently, increase the diagnostic accuracy of CRC. 

Stage-specific and location-specific diagnostic performance were also reported by several studies. Seven studies that reported location-specific performance observed lower sensitivities for CRC of the proximal colon as compared to the distal. A possible explanation is that the exfoliated colonocytes from the distal site have a shorter transit distance in the lumen, with less exposure to the cytolytic components in the gut and a greater concentration on the stool surface. As detection of CRC at its early stages (e.g., stage I, or even the precancerous lesions) is most relevant for decreasing CRC mortality, evaluation of stage-specific sensitivities is also noteworthy. However, among the 20 reviewed papers, no significant difference was found for either individual miRNA or miRNA panels for detection of early- and late-stage CRCs.

Different characteristics of study populations might potentially lead to heterogeneous diagnostic performance within reported biomarkers. Generally, study participants for diagnostic biomarker identification ought to be consistent with the target population and recruitment criteria for CRC screening. However, among all included studies, only one study [19] recruited both cases and controls from a true screening setting. Most studies recruited participants from clinical settings, which may have led to potential spectrum bias. Generally, advanced-stage cases account for higher proportions in study participants recruited from clinical setting than those from screening settings, which may lead to overestimation of sensitivity, and the applicability to the target screening population may be limited by spectrum bias [48,51]. Moreover, fecal miRNA levels might be affected by early therapeutic interventions or lifestyle modifications following diagnosis. Sixteen of 20 studies were performed on Asian populations, which might limit generalizability for other populations.

Although all miRNAs in the included studies were analyzed by qRT-PCR, substantial heterogeneity still existed regarding stool sample collection, processing, storage, and pre-analytical steps (miRNA isolation, normalization). There were, for example, great variations in time intervals (ranging from shortly to 4 days after defecation) between sample collection and storage in the laboratory freezer, with varying freezing temperatures (−80 to −20 °C) until miRNA extraction. Three reviewed studies used buffers for stool transportation to the laboratory before freezing [19,30,41], including FIT buffer and EDTA buffer. Wu et al. [23] reported that the degree of degradation of miRNAs could be reduced by using preservative buffers, such as DNA/RNA Shield, EDTA buffer, and RNAlater. It seems that miRNA markers were most stable in DNA/RNA Shield, but this result still needs further validation, as the number of stool samples was small (*n* = 5). Yamazaki et al. [52] observed that fecal miRNAs were stable in FOBT buffer for up to 5 days when stored at 4 °C [52], which would be convenient for clinical practice. Two studies [29,38] used the residual stool from FOBT analysis for miRNA measurement, and found that extracted miRNA was sufficient for further analysis.

Regarding miRNA isolation techniques, several studies found variations in the quality and quantity of miRNAs extracted from biofluids using different commercial kits. Li et al. [53] evaluated the performance of three extraction kits (Qiagen’s miRNeasy kit, Norgen’s Total RNA Purification Kit, and Ambion’s miRVana Kit), and found that the RNA isolated by Qiagen or Ambion kits had better quality than RNA isolated by the Norgen Kit. Meerson et al. [54] tested six commercial plasma isolation kits, and reported that the QIAGEN miRNeasy kits (Mini and Serum/Plasma kits) and the Macherey-Nagel NucleoSpin kit produced the greatest RNA yields from plasma compared to three other kits (Norgen Biotek Plasma/Serum kit, Zymo Research Direct-Zol Mini, and Macherey-Nagel NucleoSpin miRNA plasma). While these studies were all based on plasma samples, it is crucial to standardize the fecal miRNA extraction procedures to control differences of RNA yield that might derive from different sample preparation procedures.

Normalization is a crucial step for accurate quantitation of fecal miRNA with RT-PCR. Currently, there is no consensus on normalization methods for fecal miRNA levels. Absolute quantitation was employed in five studies for fecal miRNA [19,23,27,28,29]; however, this does not consider the influence of RNA quality on the performance of qPCR [55]. For the remaining studies, a variety of internal controls were used as references, including U6 snRNA [26,31,32,33,34,35,37,40], miR-16 [33,36], miR-200b-3p [41], cel-miR-238 [30], miR-16-3p [43], miR-1202 [39], miR-4257 [39] miR-24 [38], and miR-378 [42]. However, increasing evidence suggests that the use of internal controls for fecal miRNA detection may not be an ideal approach. For example, U6 snRNA, which is usually used as an internal control for miRNA normalization in plasma, may not be suitable for fecal miRNA detection due to its rapid degradation in stool [23]. Additionally, miRNAs selected as internal controls may also have unknown functions, and studies could be biased due to their deregulation. For example, it was reported that miR-16 could inhibit various oncogenic mRNA targets related to cancer progression [56]. Thus, a main challenge faced by many researchers is the standardization of these methodological approaches to improve the reliability and repeatability of research findings.

This systematic review provides a comprehensive up-to-date overview on fecal miRNA biomarkers for CRC detection. In addition to the studies included in previously published reviews, our study contains more recent research studies and discusses the participant characteristics, sample pre-analytics such as fecal sample collection, processing, and storage, as well as the application of different miRNA isolation kits. On account of substantial heterogeneity across the studies, we did not combine the study results in meta-analyses. Several limitations of this review need to be mentioned. Despite the comprehensive search in two well-established databases independently conducted by two reviewers, and careful cross-reference, it is still possible that relevant studies may have been missed, especially those in non-English languages, leading to language bias. Several studies were excluded because they did not report the diagnostic values, which might lead to outcome-reporting bias [57].

## 5. Conclusions

Our systematic review identified 20 studies exploring individual miRNAs, miRNA panels, or a combination of fecal miRNAs with fecal hemoglobin for CRC early detection. Fecal miRNAs for CRC detection have several advantages over other markers, such as high stability and reproducibility and the non-invasive procedure in comparison to endoscopies. However, the heterogeneity of study settings and study quality limited comparability of available evidence. Further comprehensive evaluation in large studies conducted in true screening settings is required, comparing individuals without neoplasms to participants with different adenomas or cancer stages. Promising fecal miRNA panels or their combination with other biomarkers are to be validated in large and well-designed prospective study cohorts, with standardized miRNA detection methods and minimization of pre-analytical or analytical variation.

## Figures and Tables

**Table 1:** Diagnostic performance of individual fecal miRNAs for CRC conducted in clinical settings.

First Author (Year) [Ref.]	Case-Finding Country	Cases vs. Controls		miRNA	AUC	*p*-Value	SEN (%)	SPE (%)	Validation	
		Study Group	*N*						Internal	External
Wu, C.W., et al. (2012) [23]	CS + TS Hong Kong (China)	CRC Cn	88 101	miR-21	0.64	-	56	73	-	-
				miR-92a	0.78	-	72	73		
Zhao, H.J., et al. (2014) [26]	CS China	CRC Cn	28 20	miR-194	0.74	<0.0001	60	88	-	-
Yau, T.O., et al. (2014) [27]	CS Hong Kong (China)	CRC Cn	198 198	miR-18a	0.67	-	61	69	-	-
				miR-221	0.73	-	62	74		
Wu, C.W., et al. (2014) [28]	CS Hong Kong (China)	CRC Cn	104 109	miR-135b	0.79	-	78	68	-	-
Yau, T.O., et al. (2016) [29]	CS Hong Kong (China)	CRC Cn	198 198	miR-20a	0.73	-	55	82	-	-
Chang, P.Y., et al. (2016) [30]	CS Taiwan (China)	CRC Cn	62/**76** 62/**247**	miR-223	0.79/**0.80**	<0.001	-	-	Yes	-
				miR-92a	0.79/**0.75**	<0.001	-	-		
				miR-16	0.73/**0.70**	<0.001	-	-		
				miR-20a	0.72/**0.64**	<0.001	-	-		
				miR-106b	0.71/**0.71**	<0.001	-	-		
Zhu, Y., et al. (2016) [31]	CS China	CRC Cn	80 51	miR-29a	0.78	<0.001	85	61	-	-
				miR-223	0.65	0.004	60	71		
				miR-224	0.75	<0.001	75	63		
Liu, H., et al. (2016) [32]	CS China	CRC Cn	150 98	miR-21	0.88	-	90	75	-	-
				miR-146a	0.79	-	77	68		
Li, L., et al. (2020) [33]	CS China	CRC Cn	77 29	miR-135b-5p	0.87	-	97	74	-	-
Ghanbari, R., et al. (2015) [34]	CS Iran	CRC Cn	51 26	let-7f-5p	0.71	0.003	-	-	-	-
Ghanbari, R., et al. (2015) [35]	CS Iran	CRC Cn	40 16	miR-4478	0.7	0.017	-	-	-	-
				miR-1295b-3p	0.71	0.014	-	-		
Bastaminejad, S., et al. (2017) [36]	CS Iran	CRC Cn	40 40	miR-21	0.83	-	86	81	-	-
Koga, Y., et al. (2010) [37] ^a^	CS Japan	CRC Cn	206 134	miR-17	-	-	16	89	-	-
				miR-18a	-	-	47	94		
				miR-19a	-	-	53	89		
				miR-19b	-	-	16	91		
				miR-20a	-	-	18	92		
				miR-92a	-	-	22	91		
				miR-21	-	-	15	92		
				miR-135a	-	-	15	100		
				miR-135b	-	-	46	95		
Koga, Y., et al. (2013) [38]	CS Japan	CRC Cn	117 107	miR-106a	-	-	34	97	-	-
Phua, L.C., et al. (2014) [39]	CS Singapore	CRC Cn	17 28	miR-223	0.94	-	77	96	-	-
				miR-451	0.97	-	88	100		
Choi, H.H., et al. (2019) [40]	CS Korea	CRC Cn	29 29	miR-21	0.69	-	79	48	-	-
				miR-92a	0.76	-	90	52		
				miR-144*	0.77	-	79	67		
				miR-17-3p	0.71	-	68	71		
Kalimutho, M., et al. (2011) [42]	CS Italy	CRC Cn	35 40	miR-144*	0.83	-	74	87	-	-
Rotelli, M., et al. (2015) [43] ^b^	CS Italy	CRC Cn	20 20	miR-20a-5p	0.84	-	-	-	-	-
				miR-21-3p	0.66	-	-	-		
				miR-141	0.84	-	-	-		

Note: *p*-values represent the statistical significance of AUC values; CS, collection of stools prior to any surgery or treatment from clinical settings; TS, collection of stools prior to establishment of diagnosis in a true screening setting; *N* and AUC in bold fonts represent results from the validation set (non-bold fonts represent results without validation). ^a^ Diagnostic performance was reported only for 197 fecal samples of CRC and 119 controls. ^b^ Only the values of accuracy are provided in this study. Abbreviations: Ref: Reference, *N*: number; SEN: sensitivity; SPE: specificity; AUC: area under the curve; NAA: non-advanced adenoma; CRC: colorectal cancer; Cn: control.

**Table 2:** Diagnostic performance of fecal miRNA panels for CRC.

First Author (Year) [Ref.]	Cases vs. Controls		Panel	No. of miRNA	AUC	*p*-Value	SEN (%)	SPE (%)
	Study Group	*N*						
Wu, C.W., et al. (2012) [23]	CRC Cn	88 101	Panel A	2	-	-	82	57
Yau, T.O., et al. (2014) [27]	CRC Cn	198 198	Panel B	2	0.75	-	66	75
			Panel C	2	0.78	-	66	80
			Panel D	2	0.75	-	66	75
			Panel E	3	0.79	-	71	74
Yau, T.O., et al. (2016) [29]	CRC Cn	198 198	Panel F	2	0.79	-	79	65
			Panel G	2	0.77	-	57	84
Chang, P.Y., et al. (2016) [30]	CRC Cn	62 62	Panel H	4	0.84	-	-	-
			Panel I	2	0.81	-	-	-
Liu, H., et al. (2016) [32]	CRC Cn	150 98	Panel J	2	0.88	-	87	82
Koga, Y., et al. (2010) [37] ^a^	CRC Cn	206 134	Panel K	2	-	-	46	95
			Panel L	9	-	-	74	79
			Panel M	6	-	-	70	82
Choi, H., et al. (2019) [40]	CRC Cn	29 29	Panel N	2	-	-	97	38
Wu, C.W., et al. (2017) [41]	CRC Cn	29 115	Panel O	2	0.89	<0.0001	66	95

Note: *p*-values represent the statistical significance of AUC values. Panel A: miR-21, miR-92a; Panel B: miR-18a, miR-135b; Panel C: miR-221, miR-135b; Panel D: miR-221, miR-18a; Panel E: miR-221, miR-18a, miR-135b; Panel F: miR-20a, miR-135b; Panel G: miR-20a, miR-92a; Panel H: miR-223, miR-92a, miR-16, miR-106b; Panel I: miR-223, miR-92a; Panel J: miR-21, miR-146a; Panel K: miR-135a, miR-135b; Panel L: miR-17-92 cluster*, miR-21, miR-135; Panel M: miR-17-92 cluster* (including miR-17, miR-18a, miR-19a, miR-19b, miR-20a, and miR-92a); Panel N: miR-92a, miR-144; Panel O: miR-144-5p, miR-451a; ^a^ Diagnostic performance was reported only for 197 fecal samples of CRC and 119 controls. Abbreviations: Ref: Reference; No.: number; SEN: sensitivity; SPE: specificity; AUC: area under the curve; CRC: colorectal cancer; Cn: control.

**Table 3:** Diagnostic performance of miRNAs for colorectal adenoma.

First Author (Year) [Ref.]	Case-Finding Country	Cases vs. Controls		miRNAs	AUC	*p*-Value	SEN (%)	SPE (%)
		Study Group	*N*					
Wu, C.W., et al. (2012) [23]	CS + TS Hong Kong (China)	CRA Cn	57 101	miR-92a	-	-	56	73
				Panel A	-	-	68	57
Wu, C.W., et al. (2014) [28]	CS Hong Kong (China)	AA NAA Cn	59 110 109	miR-135b	-	-	61 ^a^ 73 ^b^	68 ^a^ 68 ^b^
Yau, T.O., et al. (2016) [29]	CS Hong Kong (China)	CRA Cn	199 198	miR-20a	0.41	-	-	-
Liu, H., et al. (2016) [32]	CS China	CRA Cn	120 98	miR-21	0.77	-	85	63
				Panel J	0.76	-	79	67
Wu, C.W., et al. (2017) [41]	CS USA	AA Cn	31 115	Panel O	0.58	0.24	-	-

Note: *p*-values represent the statistical significance of AUC values; SEN, SPE, and AUC in bold fonts represent results from validation set (non-bold fonts represent results without validation); Panel A: miR-21, miR-92a; Panel J: miR-21, miR-146a; Panel O: miR-144-5p, miR-451a. ^a^ The diagnostic performance was reported for the outcome CRA (AA and NAA). ^b^ The diagnostic performance was reported for the outcome AA. Abbreviations: Ref: Reference; No.: number; SEN: sensitivity; SPE: specificity; AUC: area under the curve; NAA: non-advanced adenoma; AA: Advanced adenoma; CRA: colorectal adenoma; Cn: control.

**Table 4:** Diagnostic performance of fecal miRNAs for CRC and CRA composed of FIT-positive cases recruited from a true screening setting (*N*_CRCs/AAs/NAAs/Cns_ = 67/347/136/217).

First Author (Year) Ref.	Biomarker	Outcomes	AUC	*p*-Value	SEN (%)	SPE (%)	NPV (%)	PPV (%)
Duran-Sanchon, S., et al. (2020) [19] Duran-Sanchon, S., et al. (2021) [44]	miR-221-3p	CRC	0.70	<0.01	-	-	-	-
	miR-25-3p	CRC	0.70	<0.05	-	-	-	-
	miR-29a-3p	CRC	0.69	<0.05	-	-	-	-
	miR-34a-5p	CRC	0.71	<0.01	-	-	-	-
	miR-27a-3p	CRC	0.69	<0.05	-	-	-	-
		AA+CRC ^a^	0.63	<0.05	69	52	-	-
	miR-130b-3p	AA	0.69	<0.01	-	-	-	-
		CRC	0.71	<0.05	-	-	-	-
		AA+CRC ^a^	0.64	<0.01	82	39	-	-
	miR-421	AA	0.71	<0.001	-	-	-	-
		CRC	0.77	<0.001	-	-	-	-
		AA+CRC ^a^	0.68	<0.001	81	43	-	-
	Panel P	AA	0.71/**0.64**	-	61/**59**	71/**69**	81/**83**	47/**41**
		CRC	0.86/**0.74**	-	96/**96**	36/**33**	94/**94**	47/**41**
		AA+CRC	0.74/**0.63**	-	64/**42**	77/**73**	93/**92**	30/**15**
		AA+CRC ^a^	0.74/**0.63**		74/**67**	63/**60**	88/**85**	40/**34**
	Fecal hemoglobin	AA	0.64/**0.59**	-	50/**43**	68/**63**	72/**72**	45/**33**
		CRC	0.70/**0.67**	-	89/**100**	33/**31**	82/**100**	45/**33**
		AA+CRC	0.67/**0.59**	-	53/**45**	75/**74**	93/**92**	19/**17**
		AA+CRC ^a^	0.61/**0.62**		60/**62**	59/**58**	81/**85**	32/**27**
	miRFec	AA	0.70/**0.64**	-	50/**49**	75/**71**	56/**58**	70/**63**
		CRC	0.90/**0.93**	-	96/**97**	48/**43**	90/**94**	70/**63**
		CRC ^a^	-	-	70 ^b^	90	94	-
			-	-	85 ^b^	80	97	-
			-	-	90 ^b^	70	97	-
			-	-	93 ^b^	60	98	-
			-	-	97 ^b^	50	99	-
		AA+CRC	0.74/**0.67**	-	63/**48**	79/**75**	91/**90**	40/**22**
		AA+CRC ^a^	0.72/**0.70**	-	67/**68**	66/**64**	78/**81**	54/**47**
			-	-	30 ^b^	90	52	-
			-	-	44 ^b^	80	55	-
			-	-	56 ^b^	70	58	-
			-	-	70 ^b^	60	63	-
			-	-	79 ^b^	50	67	-
	Probability of detection and positive predictive value for AA and CRC according to the miRFec score							
	**miRFec Score**	**Odds Ratio (95% CI)**		***p*-Value**	**PPV (%)**			
	<2.14	1 ^c^		-	29			
	2.14–2.64	2.71 (1.78–4.13)		<0.001	52			
	2.65–3.09	3.56 (2.33–5.45)		<0.001	59			
	>3.09	8.08 (5.11–12.77)		<0.001	76			

Note: This study only included FIT-positive individuals in a true screening program. All results were adjusted for patient age and sex; Panel P: miR-421, miR-27a-3p, and patient age and sex; miRFec: miR-421, miR-27a-3p, fecal hemoglobin level, and patient age and sex; *p*-values represent the statistical significance of AUC values; TS, collection of stools prior to establishment of diagnosis in a true screening setting; SEN, SPE, NPV, PPV, and AUC in bold fonts represent results from the validation set (non-bold fonts represent results without validation). The validation method is 10-fold cross-validation. ^a^ Control group: patients with NAAs and Cns; ^b^ Value of the miRFec score corresponding to each specificity cut-point; ^c^ Reference category; Abbreviations: Ref: Reference, *n*: number; SEN: sensitivity; SPE: specificity; AUC: area under the curve; NPV, negative predictive value; PPV, positive predictive value; CI: confidence interval; NAA: non-advanced adenoma; AA: advanced adenoma; CRA: colorectal adenoma; CRC: colorectal cancer; Cn: control.

**Table 5:** Summary of studies reporting significant associations of miRNAs with CRC diagnosis.

Markers	Duran-Sanchon, S., et al. (2020) [19]	Wu, C.W., et al. (2012) [23]	Zhao, H.J., et al. (2014) [26]	Yau, T.O., et al. (2014) [27]	Wu, C.W., et al. (2014) [28]	Yau, T.O., et al. (2016) [29]	Chang, P.Y., et al. (2016) [30]	Zhu, Y., et al. (2016) [31]	Liu, H., et al. (2016) [32]	Li, L., et al. (2020) [33]	Ghanbari, R., et al. (2015) [34]	Ghanbari, R., et al. (2015) [35]	Bastaminejad, S., et al. (2017) [36]	Koga, Y., et al. (2010) [37]	Koga, Y., et al. (2013) [38]	Phua, L.C., et al. (2014) [39]	Choi, H., et al. (2019) [40]	Wu, C.W., et al. (2017) [41]	Kalimutho, M., et al. (2011) [42]	Rotelli, M., et al. (2015) [43]	Number of Studies
miR-21		↑○							↑○				↑△	↑○			↑△				5
miR-92a		↑○					↑○							↑○			↑○				4
miR-20a						↑○	↑△							↑○						↑△	4
miR-223							↑○	↓△								↑△					3
miR-144-5p																	↑○	↑○	↑○		3
miR-135b					↑△					↑△				↑○							3
miR-18a				↑○										↑○							2
miR-29a	↑△							↓△													2
miR-451																↑△		↑○			2
miR-221	↑△			↑○																	2
let-7f-5p											↓△										1
miR-106a															↑△						1
miR-1295b-3p												↓△									1
miR-130b-3p	↑△																				1
miR-135a														↑○							1
miR-141																				↑△	1
miR-146a									↓○												1
miR-16							↑○														1
miR-17														↑○							1
miR-17-3p																	↑△				1
miR-106b							↑○														1
miR-194			↓△																		1
miR-19a														↑○							1
miR-19b														↑○							1
miR-21-3p																				↑△	1
miR-224								↓△													1
miR-25-3p	↑△																				1
miR-27a-3p	↑○																				1
miR-34a-5p	↑△																				1
miR-421	↑○																				1
miR-4478												↓△									1

Note: Different name versions of the same miRNAs were incorporated according to the IDs of miRBase provided [45]. △ Represents markers evaluated only individually; ○ Represents markers evaluated in a panel; ↑ Represent up-regulation of markers in CRC cases compared to controls; ↓ Represent down-regulation of markers in CRC cases compared to controls.

## Supplementary Materials

The following are available online at https://www.mdpi.com/article/10.3390/cancers14010065/s1, Figure S1: Risk of bias and applicability concerns graph: review authors’ judgments about each domain presented as percentages across included studies, Figure S2: Risk of bias and applicability concerns summary: review authors’ judgments about each domain for each included study, Figure S3: Comparison of circulating miRNAs (*n* = 91) with fecal miRNAs (*n* = 31) for CRC early detection, Table S1: Participant characteristics of included fecal microRNA biomarker studies and protocols of fecal miRNA detection, Table S2: Stage-specific performance of fecal miRNAs for CRC detection, Table S3: Location-specific performance of fecal miRNAs for CRC detection, Table S4: PRISMA 2009 Checklist.
