# Supplementary material for: Fecal microRNAs, Fecal microRNA Panels, or Combinations of Fecal microRNAs with Fecal Hemoglobin for Early Detection of Colorectal Cancer and Its Precursors: A Systematic Review
Source: Cancers (Basel). 2021 Dec 23;14(1):65. doi: 10.3390/cancers14010065 (PMC8750731; doi:10.3390/cancers14010065)
Supplement: Supplementary file 1 [file cancers-14-00065-s001.zip › cancers-1492755-supplementary.pdf]

cancers

MDPI

Supplementary Materials

Fecal microRNAs, Fecal microRNA Panels, or Combinations of Fecal microRNAs with Fecal Hemoglobin for Early Detection of Colorectal Cancer and Its Precursors: A Systematic Review

Zitong Zhao, Anna Zhu, Megha Bhardwaj, Petra Schrotz-King and Hermann Brenner

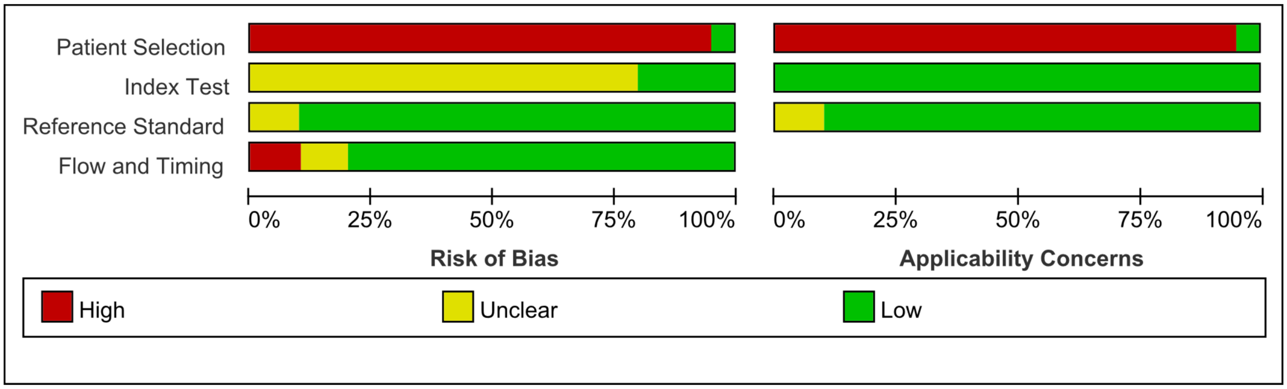

Figure S1. Risk of bias and applicability concerns graph: review authors' judgements about each domain presented as percentages across included studies.

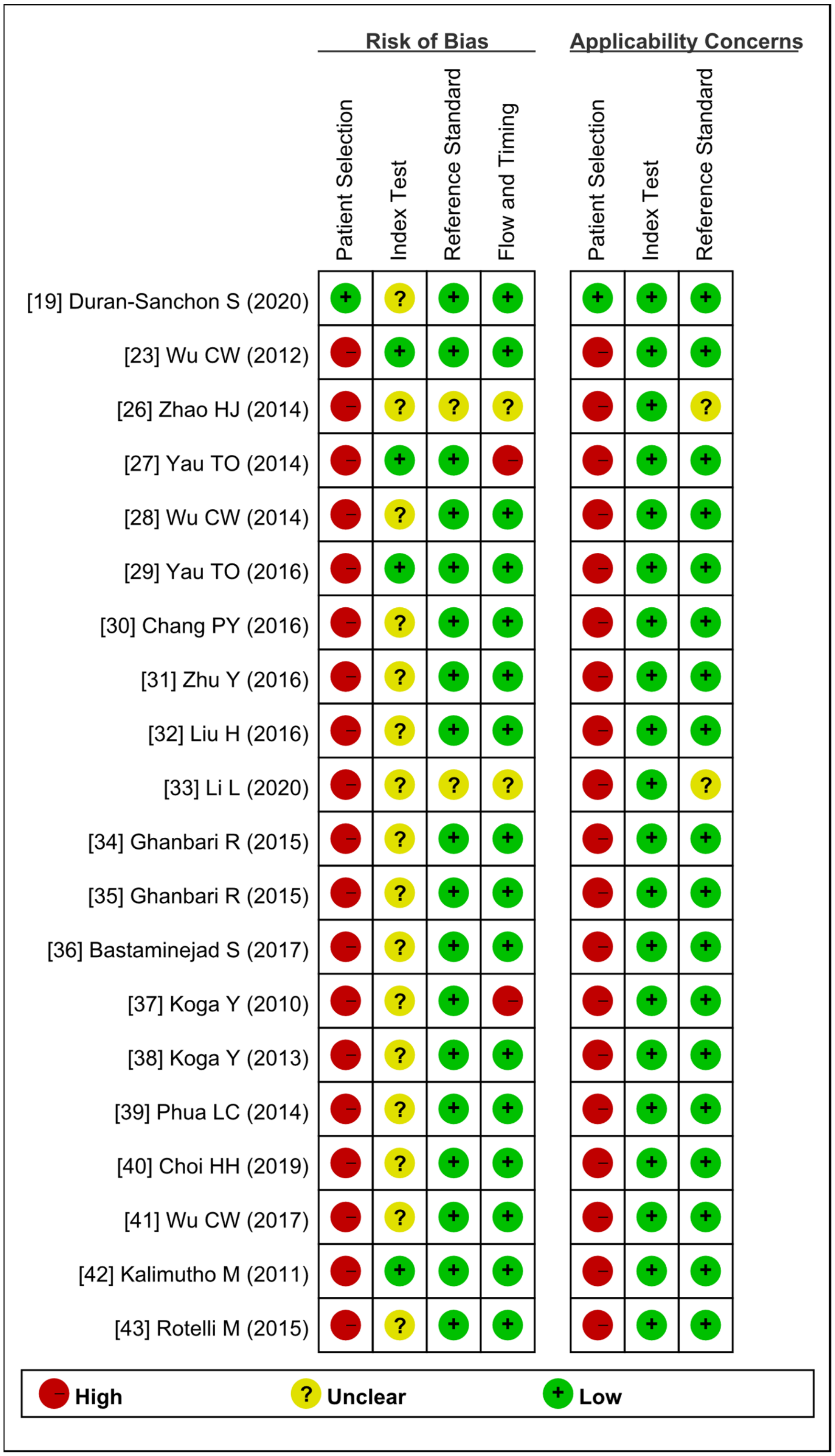

Figure S2. Risk of bias and applicability concerns summary: review authors' judgements about each domain for each included study.

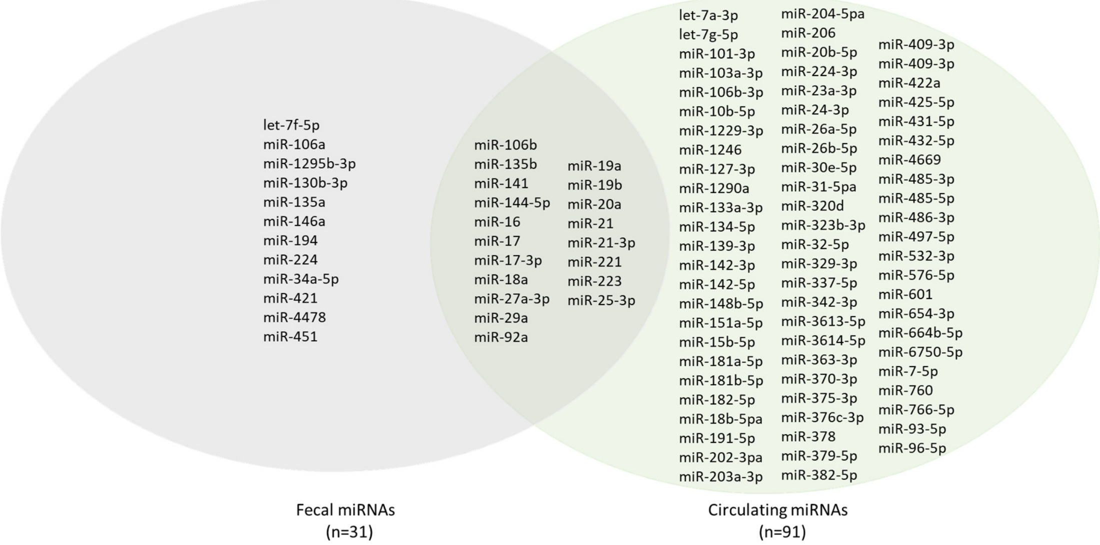

Figure S3. Comparison of fecal miRNAs (n=31) with circulating miRNAs (n=91) for CRC early detection.

Table S1. Participant characteristics of included fecal microRNA biomarker studies and protocols of fecal miRNA detection.

First author (Year)[Ref]	Case-finding country	Study group	N	Age (Mean or median)	Male (%)	CRC stage (%)		CRC location (%)		Buffer[a]	Sample storage	Sample amount	Extraction	Normalization
**Duran-Sanchon S (2020)[19]**	**TS (FIT positive) Spain**	CRC<br>AA<br>NAA<br>Cn	67<br>347<br>136<br>217	63<br>60<br>60<br>59	60<br>67<br>56<br>40	Ⅰ<br>Ⅱ<br>Ⅲ<br>Ⅳ	34<br>24<br>27<br>8	proximal<br>distal	30<br>70	FIT buffer	-80℃ [b]	500μL fecal buffers	miRNeasy mini kit (Qiagen)	Absolute
Wu CW (2012)[23]	CS + TS Hong Kong (China)	CRC<br>CRA<br>Cn	88<br>57<br>101	67<br>62<br>61	56<br>60<br>44	Ⅰ-Ⅱ<br>Ⅲ-Ⅳ	20<br>74	proximal<br>distal	31<br>69		-80℃ [a]	200-300mg feces	miRNeasy Mini Kit (Qiagen)	Absolute
Zhao HJ (2014)[26]	CS China	CRC<br>CRA<br>Cn	28<br>20<br>20	-	-	-	-	-	-	-	-	-	Trizol	U6 snRNA
Yau TO (2014)[27]	CS Hong Kong (China)	CRC<br>AA<br>CRA<br>Cn	198<br>48<br>151<br>198	67<br>59<br>60<br>59	59<br>65<br>55<br>42	Ⅰ-Ⅱ<br>Ⅲ-Ⅳ	54<br>45	proximal<br>distal<br>rectum	25<br>42<br>33	-	-80℃ [a]	200-300mg feces	miRNeasy mini kit (Qiagen)	Absolute
Wu CW (2014)[28]	CS Hong Kong (China)	CRC<br>AA<br>NAA<br>Cn	104<br>59<br>110<br>109	67<br>62<br>59<br>60	58<br>51<br>54<br>46	Ⅰ-Ⅱ<br>Ⅲ-Ⅳ	23<br>73	proximal<br>distal	28<br>72	-	-80℃ [a]	200-300mg feces	miRNeasy Mini Kit (Qiagen)	Absolute
Yau TO (2016)[29]	CS Hong Kong (China)	CRC<br>CRA<br>Cn	198<br>199<br>198	67<br>60<br>59	59<br>57<br>42	Ⅰ-Ⅱ<br>Ⅲ-Ⅳ	54<br>45	proximal<br>distal<br>rectum	25<br>42<br>33	-	-80℃ [a]	200-300mg feces	miRNeasy mini kit (Qiagen)	Absolute
Chang PY (2016)[30]	CS Taiwan (China)	CRC<br>Cn	62/**76**<br>62/**247**	62/**62**<br>61/**47**	53/**59**<br>58/**66**	Ⅰ-Ⅱ<br>Ⅲ-Ⅳ	55/-<br>45/-	-	-	FIT buffer	-80℃ [c]	300μL fecal	miRNeasy	cel-miR-238

												buff-<br>ers	mini kit<br>(Qiagen)	
Zhu Y (2016)[31]	CS<br>China	CRC<br>Cn	80<br>51	61<br>49	53<br>53	I<br>II<br>III<br>IV	14<br>34<br>37<br>15	colon<br>rectum	54<br>46	-	-80℃ [b]	200-<br>300m<br>g fe-<br>ces	stool<br>RNA ex-<br>traction<br>kit<br>(Omega)	U6<br>snRNA
Liu H (2016)[32]	CS<br>China	CRC<br>CRA<br>Cn	150<br>120<br>98	64<br>64<br>63	65<br>56<br>-	I - II<br>III-IV	45<br>55	-	-	-	-80℃ [a]	-	TRIzol	U6<br>snRNA
Li L (2020)[33]	CS<br>China	CRC<br>Cn	77<br>29	-	-	I<br>II<br>III<br>IV	14<br>12<br>30<br>44	-	-	-	Dry ice	-	Stool To-<br>tal RNA<br>purifica-<br>tion kit	miR-16,<br>U6<br>SnRNA
Ghanbari R<br>(2015)[34]	CS<br>Iran	CRC<br>Cn	51<br>26	65<br>61	53<br>54	I<br>II	24<br>76	colon<br>rectum	88<br>12	-	-80℃ [c]	100m<br>g fe-<br>ces	miR-<br>Neasy<br>mini kit<br>(Qiagen)	U6<br>snRNA
Ghanbari R<br>(2015)[35]	CS<br>Iran	CRC<br>Cn	40<br>16	63<br>60	58<br>50	I<br>II	28<br>72	colon<br>rectum	88<br>12	-	-70℃	100m<br>g fe-<br>ces	miR-<br>Neasy<br>mini kit<br>(Qiagen)	U6<br>snRNA
Bastaminejad<br>S (2017)[36]	CS<br>Iran	CRC<br>Cn	40<br>40	54<br>54	53<br>53	I<br>II<br>III<br>IV	28<br>40<br>15<br>17	colon<br>rectum	55<br>45	-	-80℃ [a]	100m<br>g fe-<br>ces	miR-<br>Neasy<br>Mini Kit<br>(Qiagen)	miR-16
Koga Y<br>(2010)[37]	CS<br>Japan	CRC<br>Cn	197<br>119	63<br>60	67<br>44	A<br>B<br>C<br>D	28<br>27<br>38<br>7	proxi-<br>mal<br>distal<br>rectum	25<br>26<br>49	-	-80℃ [c]	-	miR-<br>Neasy<br>Mini Kit<br>(Qiagen)	U6<br>snRNA
Koga Y<br>(2013)[38]	CS<br>Japan	CRC<br>Cn	117<br>107	65<br>60	59<br>62	A+B<br>C+D	65<br>35	proxi-<br>mal<br>distal	27<br>73	-	-80℃ [c]	500m<br>g fe-<br>ces	miR-<br>Neasy<br>Mini Kit<br>(Qiagen)	miR-24
Phua LC<br>(2014)[39]	CS<br>Singa-<br>pore	CRC<br>Cn	17<br>28	64<br>55	77<br>36	B<br>C<br>D	47<br>41<br>12	colon<br>rectum	47<br>53	-	-80℃ [a]	50mg<br>feces	mirVa-<br>naTM<br>miRNA<br>isolation<br>kit	miR-1202,<br>miR-4257
Choi HH<br>(2019)[40]	CS<br>Korea	CRC<br>Cn	29<br>29	67<br>53	59<br>59	I<br>II<br>III<br>IV	4<br>17<br>62<br>17	proxi-<br>mal<br>distal<br>rectum	28<br>31<br>41	-	-80℃ [b]	500m<br>g fe-<br>ces	miR-<br>Neasy<br>mini kit<br>(Qiagen)	U6<br>snRNA
Wu CW<br>(2017)[41]	CS<br>USA	CRC [d]<br>AA<br>Cn	29<br>31<br>115	61<br>65<br>66	48<br>68<br>48	I<br>II<br>III<br>IV	21<br>34<br>31<br>3	proxi-<br>mal<br>distal	55<br>45	EDTA<br>buffer	-80℃ [a]	100μL<br>fecal<br>buff-<br>ers	miR-<br>Neasy<br>mini kit<br>(Qiagen)	miR-200b-<br>3p
Kalimutho M<br>(2011)[42]	CS<br>Italy	CRC<br>Cn	35<br>40	72<br>61	43<br>33	I<br>II<br>III	6<br>17<br>17	proxi-<br>mal<br>distal<br>rectum	26<br>34<br>40	-	-20℃	200-<br>300m<br>g fe-<br>ces	mirVa-<br>na™<br>miRNA<br>isolation<br>kit	miR-378

Rotelli M (2015)[43]	CS Italy	CRC Cn	20 20	63 64	65 65	I II III	25 55 20	proxi-mal distal	40 60	-	-80℃ [a]	300mg fe-ces	miR-Neasy mini kit (Qiagen)	miR-16-3p

All miRNAs were detected using qRT-PCR; Stages I/Ⅱ/Ⅲ/Ⅳ as per Union for International Cancer Control (UICC) classification and stages A/B/C/D as per Dukes classification; Bold fonts represent results from validation set (non-bold fonts represent results without validation); CS, collection of stools prior to any surgery or treatment from clinical settings; TS, collection of stools prior to establishment of diagnosis in a true screening setting. [a] Sample were transferred to -80℃ not immediately, but within 1-4 days/store in liquid nitrogen/ kept at -20℃ freezer first/ Kept at 4℃ first; [b] Sample were transferred to -80℃ for long-term storage immediately; [c] Sample were stored at -80℃, but the transport time is unclear; Abbreviations: Ref: Reference, N: number; NAA: non-advanced adenoma; AA: advanced adenoma; CRA: colorectal adenoma; CRC: colorectal cancer; Cn: control; FIT: fecal immunochemical test; FOBT: fecal occult blood test; EDTA: ethylenediaminetetraacetic acid.

Table S2. Stage-specific performance of fecal miRNAs for CRC detection.

First author (Year)[Ref]	N (cases/controls)	miRNA	Compared groups	N	AUC	*p*-value	SEN (%)	SPE (%)
Wu CW (2012)[23a]	88/101	miR-21	Ⅰ-Ⅱ/Cn	18/101	-	1.00[b]	56	-
			Ⅲ-Ⅳ/Cn	65/101			55	
		miR-92a	Ⅰ-Ⅱ/Cn	18/101	-	1.00[b]	72	-
			Ⅲ-Ⅳ/Cn	65/101			72	
		Panel A	Ⅰ-Ⅱ/Cn	18/101	-	1.00[b]	83	-
			Ⅲ-Ⅳ/Cn	65/101			83	-
Yau TO (2014) [27a]	198/198	miR-18a	Ⅰ-Ⅱ/Cn	106/198	-	-	61	69[e]
			Ⅲ-Ⅳ/Cn	88/198		-	58	
		miR-221	Ⅰ-Ⅱ/Cn	106/198	-	-	44	74[e]
			Ⅲ-Ⅳ/Cn	88/198		-	63	
Wu CW (2014)[28]	104/109	miR-135b	Ⅰ-Ⅱ/Cn	24/109	-	-	67	68[e]
			Ⅲ-Ⅳ/Cn	76/109		-	80	
Li (2020)[33]	77/29	miR-135b-5p	Ⅰ-Ⅱ/Ⅲ-Ⅳ	20/57	0.92	0.0022[d]	81	89
			Ⅰ-Ⅲ/Ⅳ	43/34	0.78	0.0002[d]	-	-
Bastaminejad S (2017)[36]	40/40	miR-21	Ⅰ-Ⅱ/Ⅲ-Ⅳ	27/13	0.87	-	88	82
Koga Y (2010)[37]	206/134	Panel L	A+B/Cn	113/134	-	0.26[b]	71	-
			C+D/Cn	93/134			78	
		Panel M	A+B/Cn	113/134	-	0.53[b]	67	-
			C+D/Cn	93/134			72	
		miR-21	A+B/Cn	113/134	-	0.31[b]	17	-
			C+D/Cn	93/134			12	
		miR-135	A+B/Cn	113/134	-	0.20[b]	42	-
			C+D/Cn	93/134			52	
Wu CW (2017)[41a]	29/115	Panel O	Ⅰ-Ⅱ/Cn	16/115	-	0.69[c]	56	-
			Ⅲ-Ⅳ/Cn	10/115	-	-	65	

Note: Stages I/II/III/IV as per Union for International Cancer Control (UICC) classification and stages A/B/C/D as per Dukes classification; Panel A: miR-21, miR-92; Panel L: miR-17-92 cluster*, miR-21, miR-135; Panel M: miR-17-92 cluster*; Panel O: miR-144-5p, miR-451a; [a] Studies included unknown CRC stage; [b] P-values calculated using Fisher's exact test; [c] P-values calculated using chi-square test; [d] P-values represent the statistical significance of AUC values; [e] Specificity was defined by healthy controls. Abbreviations: Ref: Reference, No.: number; N: number; SEN: sensitivity; SPE: specificity; AUC: area under the curve; CRC: colorectal cancer; Cn: control.

Table S3. Location-specific performance of fecal miRNAs for CRC detection.

First author (Year)[Ref]	N (cases/controls)	Tumor Location	N	miRNAs	*p*- value	SEN (%)	SPE[c] (%)
Wu CW (2012)[23]	88/101	proximal<br>distal	27<br>61	miR-21	0.49 [a]	63.0<br>52.5	-
				miR-92a	0.01 [a]	51.9<br>80.3	-
				Panel A	0.80 [a]	70.4<br>86.9	-
Yau TO (2014)[27]	198/199	proximal<br>distal<br>rectum	50<br>82<br>66	miR-221	-	49<br>63<br>70	74[c]
				miRNA-18a	-	62<br>59<br>62	69[c]
Wu CW (2014)[28]	104/109	proximal<br>distal	29<br>75	miR-135b	> 0.05	79<br>77	68[c]
Yau TO (2016)[29]	198/199	proximal<br>distal<br>rectum	50<br>82<br>66	miR-20a	> 0.05	42<br>60<br>58	82[c]
Koga Y (2010)[37]	206/134	Right<br>Left	51<br>146	miR-135	< 0.0001 [a]	11.8<br>58.2	-
				miR-21	0.36 [a]	9.8<br>16.4	-
				Panel L	0.0001 [a]	52.9<br>81.5	-
				Panel M	0.001	51.0<br>76.0	-
Wu CW (2017)[41]	29/115	proximal<br>distal	16<br>13	Panel O	0.0084 [b]	48<br>92	95[c]

Note: Location-specific performance for distinguishing tumor at proximal/distal/rectal locations from controls; Panel A: miR-21, miR 92a; Panel L: miR-17-92 cluster*, miR-21, miR-135; Panel M: miR-17-92 cluster*; Panel O: miR-144-5p, miR-451a; [a] P-values calculated using Fisher's exact test; [b] P-values calculated using chi-square test; [c] Specificity was defined by healthy controls. Abbreviations: Ref: Reference, N: number; SEN: sensitivity; SPE: specificity; AUC: area under the curve; CRC: colorectal cancer; Cn: control.

Table S4. PRISMA 2009 Checklist.

Section/topic	#	Checklist item	Reported on page #
**TITLE**			
Title	1	Identify the report as a systematic review, meta-analysis, or both.	1
**ABSTRACT**			
Structured summary	2	Provide a structured summary including, as applicable: background; objectives; data sources; study eligibility criteria, participants, and interventions; study appraisal and synthesis methods; results; limitations; conclusions and implications of key findings; systematic review registration number.	1
**INTRODUCTION**			
Rationale	3	Describe the rationale for the review in the context of what is already known.	2
Objectives	4	Provide an explicit statement of questions being addressed with reference to participants, interventions, comparisons, outcomes, and study design (PICOS).	2
**METHODS**			
Protocol and registration	5	Indicate if a review protocol exists, if and where it can be accessed (e.g., Web address), and, if available, provide registration information including registration number.	2
Eligibility criteria	6	Specify study characteristics (e.g., PICOS, length of follow-up) and report characteristics (e.g., years considered, language, publication status) used as criteria for eligibility, giving rationale.	2-3
Information sources	7	Describe all information sources (e.g., databases with dates of coverage, contact with study authors to identify additional studies) in the search and date last searched.	2
Search	8	Present full electronic search strategy for at least one database, including any limits used, such that it could be repeated.	2
Study selection	9	State the process for selecting studies (i.e., screening, eligibility, included in systematic review, and, if applicable, included in the meta-analysis).	2-3
Data collection process	10	Describe method of data extraction from reports (e.g., piloted forms, independently, in duplicate) and any processes for obtaining and confirming data from investigators.	3
Data items	11	List and define all variables for which data were sought (e.g., PICOS, funding sources) and any assumptions and simplifications made.	3
Risk of bias in individual studies	12	Describe methods used for assessing risk of bias of individual studies (including specification of whether this was done at the study or outcome level), and how this information is to be used in any data synthesis.	3
Summary measures	13	State the principal summary measures (e.g., risk ratio, difference in means).	-
Synthesis of results	14	Describe the methods of handling data and combining results of studies, if done, including measures of consistency (e.g., I^2) for each meta-analysis.	-
Risk of bias across studies	15	Specify any assessment of risk of bias that may affect the cumulative evidence (e.g., publication bias, selective reporting within studies).	-
Additional analyses	16	Describe methods of additional analyses (e.g., sensitivity or subgroup analyses, meta-regression), if done, indicating which were pre-specified.	-
**RESULTS**			
Study selection	17	Give numbers of studies screened, assessed for eligibility, and included in the review, with reasons for exclusions at each stage, ideally with a flow diagram.	3, Figure 1
Study characteristics	18	For each study, present characteristics for which data were extracted (e.g., study size, PICOS, follow-up period) and provide the citations.	3-4, Table S1
Risk of bias within studies	19	Present data on risk of bias of each study and, if available, any outcome level assessment (see item 12).	6, Figures S1-S2

Results of individual studies	20	For all outcomes considered (benefits or harms), present, for each study: (a) simple summary data for each intervention group (b) effect estimates and confidence intervals, ideally with a forest plot.	3-6, Tables 1-4, Tables S1-S3
Synthesis of results	21	Present results of each meta-analysis done, including confidence intervals and measures of consistency.	-
Risk of bias across studies	22	Present results of any assessment of risk of bias across studies (see Item 15).	-
Additional analysis	23	Give results of additional analyses, if done (e.g., sensitivity or subgroup analyses, meta-regression [see Item 16]).	-
**DISCUSSION**			
Summary of evidence	24	Summarize the main findings including the strength of evidence for each main outcome; consider their relevance to key groups (e.g., healthcare providers, users, and policy makers).	11-13
Limitations	25	Discuss limitations at study and outcome level (e.g., risk of bias), and at review-level (e.g., incomplete retrieval of identified research, reporting bias).	13
Conclusions	26	Provide a general interpretation of the results in the context of other evidence, and implications for future research.	13
**FUNDING**			
Funding	27	Describe sources of funding for the systematic review and other support (e.g., supply of data); role of funders for the systematic review.	13

References.

19. Duran-Sanchon S, Moreno L, Auge JM, et al. Identification and Validation of MicroRNA Profiles in Fecal Samples for Detection of Colorectal Cancer. Gastroenterology. 2020;158(4):947-957.e944.
23. Wu CW, Ng SS, Dong YJ, et al. Detection of miR-92a and miR-21 in stool samples as potential screening biomarkers for colorectal cancer and polyps. J Gut. 2012;61(5):739-745.
26. Zhao HJ, Ren LL, Wang ZH, et al. MiR-194 deregulation contributes to colorectal carcinogenesis via targeting AKT2 pathway. Theranostics. 2014;4(12):1193-1208.
27. Yau TO, Wu CW, Dong Y, et al. microRNA-221 and microRNA-18a identification in stool as potential biomarkers for the non-invasive diagnosis of colorectal carcinoma. Br J Cancer. 2014;111(9):1765-1771.
28. Wu CW, Ng SC, Dong Y, et al. Identification of microRNA-135b in stool as a potential noninvasive biomarker for colorectal cancer and adenoma. Clinical cancer research : an official journal of the American Association for Cancer Research. 2014;20(11):2994-3002.
29. Yau TO, Wu CW, Tang CM, et al. MicroRNA-20a in human faeces as a non-invasive biomarker for colorectal cancer. Oncotarget. 2016;7(2):1559-1568.
30. Chang PY, Chen CC, Chang YS, et al. MicroRNA-223 and microRNA-92a in stool and plasma samples act as complementary biomarkers to increase colorectal cancer detection. Oncotarget. 2016;7(9):10663-10675.
31. Zhu Y, Xu A, Li J, et al. Fecal miR-29a and miR-224 as the noninvasive biomarkers for colorectal cancer. Cancer Biomarkers. 2016;16(2):259-264.
32. Liu H, Gong W, Lou J, et al. MicroRNA-21 and microRNA-146a identification in stool and its clinical significance in colorectal neoplasms. International Journal of Clinical and Experimental Medicine. 2016;9(8):16441-16449.
33. Li L, Wang A, Cai M, Tong M, Chen F, Huang L. Identification of stool miR-135b-5p as a non-invasive diaognostic biomarker in later tumor stage of colorectal cancer. Life sciences. 2020;260.
34. Ghanbari R, Mosakhani N, Sarhadi VK, et al. Simultaneous Underexpression of let-7a-5p and let-7f-5p microRNAs in Plasma and Stool Samples from Early Stage Colorectal Carcinoma. Biomarkers in cancer. 2015;7(Suppl 1):39-48.
35. Ghanbari R, Mosakhani N, Asadi J, et al. Decreased expression of fecal miR-4478 and miR-1295b-3p in early-stage colorectal cancer. Cancer Biomarkers. 2015;15(2):189-195.
36. Bastaminejad S, Taherikalani M, Ghanbari R, Akbari A, Shabab N, Saidijam M. Investigation of MicroRNA-21 Expression Levels in Serum and Stool as a Potential Non-Invasive Biomarker for Diagnosis of Colorectal Cancer. Iranian biomedical journal. 2017;21(2):106-113.

37. Koga Y, Yasunaga M, Takahashi A, et al. MicroRNA Expression Profiling of Exfoliated Colonocytes Isolated from Feces for Colorectal Cancer Screening. Cancer Prevention Research. 2010;3(11):1435-1442.
38. Koga Y, Yamazaki N, Yamamoto Y, et al. Fecal miR-106a Is a Useful Marker for Colorectal Cancer Patients with False-Negative Results in Immunochemical Fecal Occult Blood Test. Cancer Epidemiology Biomarkers & Prevention. 2013;22(10):1844-1852.
39. Phua LC, Chue XP, Koh PK, Cheah PY, Chan EC, Ho HK. Global fecal microRNA profiling in the identification of biomarkers for colorectal cancer screening among Asians. Oncol Rep. 2014;32(1):97-104.
40. Choi HH, Cho YS, Choi JH, Kim HK, Kim SS, Chae HS. Stool-Based miR-92a and miR-144* as Noninvasive Biomarkers for Colorectal Cancer Screening. Oncology. 2019;97(3):173-179.
41. Wu CW, Cao X, Berger CK, et al. Novel Approach to Fecal Occult Blood Testing by Assay of Erythrocyte-Specific microRNA Markers. Digestive Diseases and Sciences. 2017;62(8):1985-1994.
42. Kalimutho M, Blanco GDV, Di Cecilia S, et al. Differential expression of miR-144* as a novel fecal-based diagnostic marker for colorectal cancer. Journal of Gastroenterology. 2011;46(12):1391-1402.
43. Rotelli MT, Di Lena M, Cavallini A, et al. Fecal microRNA profile in patients with colorectal carcinoma before and after curative surgery. International Journal of Colorectal Disease. 2015;30(7):891-898.
